# Unintentional childhood injury: a controlled comparison of behavioral characteristics

**DOI:** 10.1186/s12887-016-0558-1

**Published:** 2016-01-29

**Authors:** Hui Zhang, Yang Li, Yuxia Cui, Hongling Song, Yong Xu, Shih-Yu Lee

**Affiliations:** School of Nursing, Harbin Medical University (Daqing), No. 39 Xinyang Road, Gaoxin District, Daqing City, Hei Longjiang Province 163319 China; Department of Nursing, Hungkaung University, No. 1018, Sec. 6, Taiwan Boulevard, Shalu Dist, Taichung, 43302 Taiwan ROC; English Department, Harbin Medical University (Daqing), No. 39 Xinyang Road, Gaoxin District, Daqing, Hei Longjiang Province 163319 China; ICU, Daqing People’s Hospital, No. 213 Jianshe Road, Gaoxin District, Daqing City, Hei Longjiang Province 163316 China

**Keywords:** Unintentional injury, Children, Risk behavior, CBCL, Behavior disorder predictors

## Abstract

**Background:**

Childhood injury is a major public health problem around the world and those injuries have negative impacts on children and their families. The purpose of this study was to compare the behavioral characteristics between Chinese school-age children (6 to 11 years of age) with and without unintentional injuries and to identify behavioral risk factors for school-age children with unintentional injury.

**Methods:**

This cross-sectional predictive study was conducted in five elementary schools in Daqing, China. The Achenbach Child Behavior Checklist (CBCL) was used to assess the children’s behavioral characteristics. A total of 725 school-age children were screened. Of these, 116 children who had experienced unintentional injury in the past year were recruited as the study group, and 123 children who had not experienced an unintentional injury were randomly selected and assigned to the control group.

**Results:**

The total scores of CBCL in the study group children were significantly higher than those in the control group. The significant behavior disorder predictors for unintentional injury in boys were schizoid behavior problem (OR = 2.43), anxiety/depression (OR = 2.76) and hyperactive (OR = 2.42). The predictors for unintentional injury in girls were anxiety/depression (OR = 2.12) and delinquent behavior (OR = 2.81).

**Conclusions:**

Children with behavior disorders are more likely to suffer from unintentional injuries. Teachers and pediatricians should identify the behavior disorders and assist parents to help children, thereby reducing the rate and severity of injuries.

## Background

Childhood injury is a major public health problem around the world [1]. Over 90 % of injuries to children occur in low- and middle-income countries [2]. In Chinese society, unintentional injuries are the most common cause of morbidity and mortality for children under age 14, and those injuries have negative impacts not only on children but also on their families [3].

An unintentional injury is a fatal or non-fatal physical injury that occurs suddenly [4]. The prevalence rate of unintentional injury in China ranges from 11.3 to 13.9 % among children who had medically attended injuries before age 14 [3, 5, 6]. Falls, burns, and motor vehicle crash are the most common types of childhood injury [7]. The mortality rate for unintentionally injured children under 14 is about 0.7 % and accounts for 31.3 % of total child deaths in China [8]. In Beijing China, more than 10 % of children under age 14 required medical care for injuries in 2003, and the annual medical cost was at least ¥82 million (about US $14 million) [9]. The burdens of pediatric injury may overload the families of the injured children and may put an indirect burden on society as well. There are no national cost statistics available for China as a whole; however, in Guangdong Province, medical costs for disability care and non-routine medical treatment for elementary and middle school students between 1998 and 1999 have been estimated at about ¥369 million (about $62 million) [10].

Previous studies have identified that unintentional injuries in children are associated with socioeconomic and environmental factors, including poverty, low education level of parents, young age of mother, unemployed/underemployed father, poor parental supervision, and unsafe utilities at home or playground [11–13]. The characteristics of the affected children are also associated with the prevalence of unintentional injuries. For example, boys who have experienced injury tend to have a difficult type of temperament, a lower ability to concentrate on homework, greater academic stress, and various behavior disorders [14–16].

Although children may be injured in a variety of different places, studies reveal that unintentional injury tends to occur more often at home for toddlers and preschoolers, while elementary school children are more likely to be injured outdoors [17, 18]. The explanation for this may be related to exposure (in the home vs. outdoors) [19]. Some studies that have focused on the association between behavior disorders (e.g., hyperactivity, aggression, anxiety) and injury, most have focused on preschoolers; few studies have paid attention to these factors in school-age children [20]. Therefore, the aim of this study was to compare differences in behavioral characteristics between Chinese 6 to11 year old/1^st^ to 5^th^ grade school-age children who sustained a non-fatal unintentional injury in the previous year and children who did not sustain an injury. That data were further used to identify risk behavior factors for the injured children.

## Methods

### Definition of Non-fatal Unintentional Injury

Based on ICD-10 [21] a non-fatal unintentional injury was operationally defined as an injury that (a) was diagnosed as an injury by physicians and received medical treatment or (b) was not diagnosed but because of traffic accident, drowning, choking, poisoning, burns, falling, animal biting or suicide/homicide, the children received emergent medical assistance from adults (teachers, parents or others) and (c) required the child to rest for more than half a day before returning to normal activity [22].

### Study design and participants

A cross-sectional predictive study was conducted at five elementary schools (1^st^ to 5^th^ grade and 6 to 11 years old children) in the city of Daqing, in the northeast region of China. Data were collected from the regular parents’ meetings in school, either at the beginning or the end of fall semester from September 2012 to January 2013. Data were obtained from the children’s primary caregivers.

### Questionnaire

Demographic variables included parents’ age, education, marital status, child’s age and gender, family type (nuclear, extended, single parent), and annual household income. Data on these variables were collected on the sociodemographic form.

### Unintentional Injury Screening Tool

An Unintentional Injury Screening Tool was developed by the researcher based on recent Chinese epidemiology data and a literature [9, 22]. The tool was used to screen potential study participants. The primary caregivers were asked whether their child had experienced a non-fatal unintentional injury in the previous 12 months, whether the child had received medical and other treatments, and whether the child was required to rest for more than half a day because of the injury.

### The Achenbach Child Behavior Checklist (CBCL)

The CBCL is a widely used, empirically derived measure of children’s behavioral problems [23]. It is a 113-item, 3-point Likert scale given to parents to assess the behavior disorders of their children in the previous 12 months. The CBCL has been translated into a Chinese version and tested in Chinese children [24]. The scoring system is gender based and has different cut-off points for each gender. A higher score indicates more behavior disorders. The subscales are different between genders (see details in Tables 2 and 3) and have various cut-off points [24]. A behavioral disorder is considered to exist when the mean score exceeds the cut-off point in any of the subscales [24]. In this study, the Cronbach’s α was 0.98 for the whole scale and above 0.7 for all the subscales in both genders, with an exception for the aggressive subscale for boys that was 0.43. However, after deleting item 94 (teases a lot) from the CBCL, the Cronbach’s α was increased to 0.94, therefore that item was excluded for the rest of data analysis for boys.

### Procedure

All data were obtained from the children’s primary caregivers. Three-step sampling was used. There are four to six classes per grade at the elementary schools we recruited from. First, two to three classes (about 40 students in each class) from each grade were randomly selected from each school using a lottery. A total of 725 children from the five schools, along with their primary caregivers, were then invited to fill out the questionnaires (describe later). Children with attention deficit hyperactivity disorder (ADHD), autism, and schizophrenia were excluded from this study because we intended to generalize the findings to healthy school-age children. Children with autism and schizophrenia were automatically excluded from this study because they have to attend special school according to the regulation in China. The Child Behavior Rating Scale (CBRS-teachers) was used to screening potential study participants for ADHD [25]; those who scored ≥10 were further evaluated by a psychiatrist to rule out ADHD.

Informed and verbal consents were obtained from all primary caregivers. The researcher verbally explained the non-fatal unintentional injury definition to the parents before data collection; a written definition was also provided on the questionnaire to reinforce what non-fatal unintentional injury is. The caregivers filled out the questionnaire at home and gave it to their children in a sealed envelope to return to the classroom teacher, the response rate was 100 %. The primary investigator then picked up the envelope. Second, children aged 6–11 years who had experienced a non-fatal unintentional injury in the previous 12 months were selected and assigned to the study group. Finally, using a pre-prepared list of random numbers, a comparison group of uninjured children (the control group) was selected from among the other children who hadn’t experience the non-fatal unintentional injury, to match control-group children by gender and age with the children in the study group.

### Data analyses

All data were analyzed by using SPSS Version 18.0. The questionnaire was excluded if it had more than 20 % missing data. The categorical variables were described as frequency and percentage. The differences between the two groups were compared using the cross tabulation analysis and T-tests. Continuous variables were described as mean and standard deviation (SD). Spearman’s correlation was used to explore the association between the incidence of unintentional injury and each CBCL subscale. T-tests were used to compare the differences of CBCL scores between the study group and control group. After controlling for different sociodemographic variables between the two groups, logistical regression analysis was performed to identify the behavioral predictors for unintentional injury, with the total and subscale scores of CBCL as independent variables and the occurrence of unintentional injury as the dependent variable.

### Ethics statement

Ethics approval was obtained from Harbin Medical University. Verbal and written consents were obtained from primary caregivers as a pre-requisite to collecting information and required an explanation of the research project, what it consisted of, and the type of data being collected.

## Results

### Participant characteristics

Among the 725 children (375 boys and 350 girls), the response rate was 100 %. A total of 130 children (17.9 %) met the inclusion criteria and were recruited into the injury group; however, 14 children were excluded because their primary caregiver questionnaires had more than 20 % missing data; thus, the valid response rate was 89.2 %. A total of 595 children hadn’t experience unintentional injury in the past 12 months, and 5 children were excluded because their questionnaires had more than 20 % missing data. Among these 590 children, 123 control group children were selected by using a pre-prepared list of random numbers. The comparison of demographic characteristics between the two groups and genders is detailed in Table 1. Among the 116 children in the injury group, 69 were boys (59.5 %) and 47 were girls (40.5 %) with a mean age of 8.06 (SD = 0.94). The injury incidence rate for boys was 9.5 % and 6.5 % for girls, but the rate showed no statistically significant difference between genders (*p* = 0.815). The mother’s education, marital status of family, and relationship between caregiver and child had significant differences between injury boys and control boys. Parents in the injury group had a significantly higher education level than those in the control group (*p* < 0.01). The places where the injuries were most likely to occur were school, home, playground, and street. The majority of primary caregivers in the injury group had at least a college level education (>51 %), and typical family type was a nuclear family (68.5 %). The control group had 123 children, including 75 boys (61 %) and 48 girls (39 %) with a mean age of 8.03 (SD = 1.67). About 60 % of the control group parents were educated at the middle-school level, and about half (52.5 %) reported living in a nuclear family (52.5 %).

**Table 1:** Demographic characteristics of children and their families for the injury and control groups

Variables	Boys			Girls		
	Injury group	Control group	*p*-value	Injury group	Control group	*p*-value
	(*n* = 69)	(*n* = 75)		(*n* = 47)	(*n* = 48)	
Child mean age^a^	8.05 ± 0.21	7.93 ± 1.74	0.671	8.06 ± 1.67	8.12 ± 1.59	0.705
Mother’s education^b^			<0.01			0.011
Primary school and below	1(1.4 %)	0		0	0	
Middle school	33(47.8 %)	65(86.7 %)		20(42.6 %)	9(18.8 %)	
College and above	35(50.7 %)	10(13.3 %)		27(57.4 %)	39(81.3 %)	
Father’s education^b^			<0.01			<0.01
Primary school and below	1(1.4 %)	0		1(2.1 %)	0	
Middle school	30(43.5 %)	70(93.3 %)		24(50.1 %)	6(12.5 %)	
College and above	38(55.1 %)	5(6.7 %)		22(46.8 %)	42(87.5 %)	
Marital status of family^b^			0.013			0.096
Married	63(91.3 %)	74(98.6)		44(93.6 %)	46(95.8 %)	
Divorced/Single	3(4.3 %)	1(1.4 %)		2(4.3 %)	1(2.1 %)	
Remarried	3(4.3 %)	0		1(2.1 %)	1(2.1 %)	
Family Type^b^			0.083			0.057
Single-parent family	3(4.3 %)	1(1.3 %)		2 (4.3 %)	1(2.1 %)	
Nuclear family	49(71 %)	64(85.3 %)		31(66 %)	41(85.4 %)	
Extended family	17(24.7 %)	10(13.4 %)		13(27.7 %)	6(12.5 %)	
Primary caregiver for child^b^			0.024			0.111
Parent(s)	45(66.7 %)	55(73.3 %)		35(74.5 %)	36(75 %)	
Grandparent(s)	13(18.8)	5(6.7 %)		9(19.1 %)	3(6.3 %)	
Babysitter	3(4.3 %)	0		1(2.1 %)	3(6.3 %)	
Other	8(10.2 %)	15(20 %)		2(4.3 %)	6(12.4 %)	
Household income (per person per month in yuan)^b^			0.369			0.081
<1000	8(11.6 %)	10(13.3 %)		12(25.5 %)	3(6.3 %)	
1000–3000	23(33.3)	30(40 %)		14(34 %)	18(37.5 %)	
3000–5000	32(46.4 %)	30(40 %)		16(29.8 %)	24(50 %)	
>5000	6(8.7 %)	5(6.7 %)		5(10.6 %)	3(6.3 %)	
Place of injury^b^						
School	26(37.5 %)			13(28.6 %)		
Home	23(32.7 %)			19(39.7 %)		
Playground	11(15.4 %)			6(12.7 %)		
Street	10(14.4 %)			9(19 %)		

Note: ^a^The comparison of variable (age) was done using t-test, ^b^The comparison of categorical variables including (Mother’s education, Father’s education, Marital status of family, Family Type, Caregiver for child, Household income) were done the cross tabulation analysis

### Behavioral characteristics of children with and without unintentional injury

The injury group children had a significantly higher CBCL score compared to those in the control group for both genders (*p* < 0.01). The distribution of CBCL scores was skewed but normalized after transformation; therefore, independent t-tests were used for further comparison. Compared to the control group, both boys and girls in the injury group scored a significantly higher level of behavior disorder problems (*p* < 0.001) in all behavioral types measured in the CBCL (see Tables 2 and 3). The externalizing behavior and internalizing behavior of the injury group were higher than the control group. Children who scored above the cut-off point in any subscale were categorized as having a behavioral disorder [24]. The behavior disorder prevalence rates were 33.3 % (23/69) for boys and 40.4 % (19/47) for girls in the injury group and much lower at 6.67 % (5/75) for boys and 8.3 % (4/48) for girls in the control group.

**Table 2:** Comparison of scores for CBCL between injury and control group (boys)

Behavior Subscale (cut-off)	Whole (*n* = 144)		Injury group (*n* = 69)			Control group (*n* = 75)			t	*p-*value
	Mean	SD	Mean	SD	Range	Mean	SD	Range		
Schizoid (5–6)	2.49	2.99	3.90	3.62	(0–12)	1.20	1.28	(0–4)	-5.86	0.000
Anxiety/Depression (9–10)	4.08	5.92	6.64	7.30	(0–23)	1.73	2.69	(0–10)	-5.27	0.000
Social problems (5–6)	2.31	3.14	3.43	4.05	(0–16)	1.27	1.30	(0–4)	-4.25	0.004
Compulsive activity(8–9)	3.66	5.33	5.97	6.90	(0–23)	1.53	1.32	(0–5)	-5.26	0.000
Somatic complaints (6–7)	1.90	3.05	3.32	3.83	(0–12)	0.60	0.96	(0–3)	-5.73	0.000
Social withdrawal(5–6)	1.98	2.91	3.04	3.53	(0–13)	1.00	1.69	(0–6)	-4.37	0.000
Hyperactivity (10–11)	4.02	3.80	5.28	4.66	(0–17)	2.87	2.26	(0–9)	-3.89	0.007
Aggressive behavior (19–20)	6.57	7.50	9.36	9.19	(0–33)	4.00	4.14	(0–17)	-4.45	0.001
Delinquent behavior (7–8)	2.43	4.36	4.42	5.53	(0–18)	0.60	1.26	(0–4)	-5.60	0.000
Internalizing behavior	14.44	19.34	23.3	24.6	(0–75)	6.33	5.42	(1–20)	-5.60	0.000
Externalizing behavior	13.02	15.11	19.1	18.8	(0–68)	7.47	7.10	(1–27)	-4.81	0.000
Total score(40–42)	30.86	37.76	47.45	47.8	0–145	15.60	12.61	3–53	-5.36	0.000

**Table 3:** Comparison of scores for CBCL between injury group and control group (girls)

Behavior Subscale (cut-off)	Whole (*n* = 144)		Injury group (*n* = 69)			Control group (*n* = 75)			t	*p-*value
	Mean	SD	Mean	SD	Range	Mean	SD	Range		
Schizoid (5–6)	2.49	2.99	3.90	3.62	(0–12)	1.20	1.28	(0–4)	-5.86	0.000
Anxiety/Depression (9–10)	4.08	5.92	6.64	7.30	(0–23)	1.73	2.69	(0–10)	-5.27	0.000
Social problems (5–6)	2.31	3.14	3.43	4.05	(0–16)	1.27	1.30	(0–4)	-4.25	0.004
Compulsive activity (8–9)	3.66	5.33	5.97	6.90	(0–23)	1.53	1.32	(0–5)	-5.26	0.000
Somatic complaints (6–7)	1.90	3.05	3.32	3.83	(0–12)	0.60	0.96	(0–3)	-5.73	0.000
Social withdrawal (5–6)	1.98	2.91	3.04	3.53	(0–13)	1.00	1.69	(0–6)	-4.37	0.000
Hyperactivity (10–11)	4.02	3.80	5.28	4.66	(0–17)	2.87	2.26	(0–9)	-3.89	0.007
Aggressive behavior (19–20)	6.57	7.50	9.36	9.19	(0–33)	4.00	4.14	(0–17)	-4.45	0.001
Delinquent behavior (7–8)	2.43	4.36	4.42	5.53	(0–18)	0.60	1.26	(0–4)	-5.60	0.000
Internalizing behavior	16.6	18.7	24.2	21.1	(0–62)	9.1	12.1	(0–48)	-4.27	0.000
Externalizing behavior	11.9	14.3	17.7	15.5	(0–49)	6.3	10.2	(0–42)	-4.22	0.000
Total score (40–42)	30.86	37.76	47.45	47.8	0–145	15.60	12.61	3–53	-5.36	0.000

### Behavioral predictors for unintentional injury

Unintentional injury was significantly associated with all the behavior disorder types measured in the CBCL for both genders (*r*_*s*_ = 0.241-0.433, *p* < 0.05). It was also associated with parent characteristics, such as education level and marriage status. After controlling for sociodemographic factors, schizoid behavior problem (OR = 2.43, 95 % CI = 1.44-4.11), anxiety/depression (OR = 2.76, 95 % CI = 1.50-5.06), and hyperactivity (OR = 2.42, 95 % CI = 1.26-4.68) were determined to be the predictors for injury in boys. The results indicate that boys who had scores above the cut-off in schizoid, anxiety/ depression, and hyperactivity behavior were 2.42 to 2.76 times more likely to have an injury than boys with normal scores. For girls, anxiety/depression (OR = 2.12, 95 % CI = 1.97-4.64) and delinquent behavior (OR = 2.81, 95 % CI = 1.41-4.61) were predictors of injury. The results indicate that girls with scores above the cut-off in anxiety/depression and delinquent behaviors were 2.12 to 2.81 times more likely to suffer an injury than girls with normal scores (see Table 4).

**Table 4:** Logistic regression analysis of CBCL and unintentional injury

Variables		B	SE	Wald	OR	95 % CI for OR		*p-*value
						Lower	Upper	
Boys	Schizoid	0.887	0.269	10.916	2.428	1.435	4.11	0.001
	Anxiety/Depression	1.014	0.310	10.695	2.756	2.521	5.06	0.013
	Social problems	-0.153	0.339	0.203	0.859	0.442	1.668	0.653
	Compulsive activity	0.360	0.358	1.013	1.434	0.711	2.892	0.314
	Somatic complaints	-0.141	0.369	0.146	0.868	0.421	1.791	0.702
	Social withdrawal	-0.375	0.496	0.570	0.687	0.260	1.819	0.450
	Hyperactivity	0.873	0.245	12.661	2.418	1.258	4.68	0.001
	Aggressive behavior	-0.531	0.267	3.937	0.589	0.349	0.994	0.052
	Delinquent behavior	1.080	1.198	0.813	1.091	0.581	2.047	0.787
Girls	Schizoid	0.171	0.167	1.045	1.187	0.855	1.648	0.307
	Anxiety/Depression	0.752	0.399	10.545	2.121	1.970	4.64	0.001
	Social problems	0.278	0.205	1.837	1.321	0.883	1.974	0.175
	Compulsive activity	-0.319	0.311	1.050	0.727	0.395	1.337	0.306
	Somatic complaints	-0.121	0.174	0.481	0.886	0.630	1.247	0.488
	Social withdrawal	0.047	0.309	0.023	1.048	0.572	1.920	0.879
	Hyperactivity	0.689	0.383	3.239	1.991	0.941	4.215	0.072
	Aggressive behavior	0.027	0.094	0.084	1.028	0.854	1.236	0.772
	Delinquent behavior	1.206	0.348	11.352	2.813	1.411	4.61	0.000

Note: *OR* odds ratio, *CI* confidence interval

## Discussion

Results from this study showed that the incidence of unintentional injury for the group as a whole was 17.9 %, which is higher than the earlier reports for children aged 14 and under in China [3, 5]. Previous studies showed that boys experience injury more frequently than girls in all age groups [26, 27]; however, there was no statistically significant difference between boys (9.5 %) and girls (6.5 %) in this study (*p* = 0.815), though the boys did have a higher incidence of injury than girls. In the present study, injury often happened either at home or at school, which differed from the findings of previous studies where injuries often occurred in a public place [17, 18, 28]. One reason for the difference may lie in differences in the nature of the children’s activity. In this study, boys spent more time using the computer at home, thus decreasing the outdoor risk exposure [29]. Also, in this study, parents in the boys’ injury group had a statistically significant higher education level compared to the control group parents, which was inconsistent with other researchers’ findings [11–13]. The higher incidence of injury might be due to higher-educated parents having less time with their children because of work commitments that kept them away from home. These working parents may have been compelled to leave their children in higher risk environments for longer periods than lower-educated parents, who were more likely to be at home directly supervising their children [30].

Findings from this study indicate that children with an unintentional injury have higher scores in CBCL than those without injury (*p* < 0.01). The findings are consistent with recent evidence that children with more (and more severe) behavior disorders are more likely to suffer injury [13, 26, 31]. In general, behavioral disorders are common in school-age children; during this phase of life, children have stable physical development but are more likely to have emotional problems and impulsive behaviors, which could cause them to engage in careless and risky behaviors [32]. School-age children spend lots of time at school, and the faculty members are responsible for their supervision during school hours. However, in China, the ratio between teachers and students is about 1:40 ~ 50, which was highlighted as the main cause for injuries in a previous study [32]. Researchers point out that most children with an unintentional injury had emotional instability when they encountered a hazardous environment [13, 33]. The current study had similar findings, namely that children with more behavior disorders tended to have more injury incidents. Interestingly, girls had more behavior disorders (40.4 %) compared to boys (33.3 %) in the current study; this could be the result of different parenting patterns between boys and girls because Chinese boys are more often punished by their parents when they make mistakes [34–37]. In Chinese culture, parents tend to strictly discipline boys for mistakes or behavior problems, whereas these behaviors would be tolerated (or even rewarded) in girls. This might have the effect of reinforcing behavior disorders in girls [38]. An alternative explanation for this phenomenon could be related to the different scoring systems between genders and the fact that there are higher cut-off points for boys in the CBCL. The appropriateness of using a gender-based scoring system for the CBCL may need to be further explored in Chinese society.

Logistic regression analysis indicates that schizoid, anxiety/depression, and hyperactivity are the significant predictors for injury in boys, while anxiety/depression and delinquent behavior are the predictors for girls. Boys and girls with the above behavior disorders had more than two times greater likelihood of experiencing an unintentional injury than those children who had normal CBCL scores. Anxiety/ depression was a predictor of injury for both boys and girls. This result is different from previous research that showed children with externalizing behavior are more likely to be impulsive and are at great risk for injury [39–41]. However, a recent study revealed that Chinese children with depression or delinquent behaviors were also inclined to have risk behaviors and injuries later in life, including substance abuse, aggression, and suicide [42]. In China, anxiety and depression are very common among school-age children, which could result from a tendency of Chinese parents to overemphasize academic success [43, 44]. For example, a recent study of the effects of stress on school-age children (*N* = 2,191) indicated that one third of Chinese children who experience psychological problems, particularly anxiety and depression, as a result of academic requirements and parental pressure, are inclined to have injury episodes at home and at school [45].

The current study found that unintentional injuries happened most often at school and at home. This suggests that parents, school teachers, and pediatric health care providers should pay more attention to children who have behavior problems, especially those with hyperactivity, anxiety/depression, and delinquent behaviors. To prevent injury, it is necessary for schools and community health centers to screen children’s behaviors on a regular basis, and parents should be encouraged to seek help when they notice problem behaviors in their youngsters.

This study contributes knowledge to the Chinese medical community on the association between children’s behavioral characteristics and injury events. Specifically, it reveals significant behavioral predictors for injury in school-age children. However, the findings should be considered in light of several methodological limitations. First, children’s behaviors were assessed in the context of unintentional injuries that had occurred within the previous 12 months. Recall bias could occur, in particular for identifying minor injuries because they might be easily forgot, thus threatening the study’s internal validity; therefore, a prospective study is needed. Second, the participants of this study were all mentally healthy children. Children with ADHD and who could be more inclined to have unintentional injuries were excluded, but should be included in future studies to provide a more complete explanation. Third, data on the severity of unintentional injuries was not collected in this study, so the association, if any, between severity of injury and behavior problems could not be determined. Last, the participants were all from five elementary schools in one city (Daqing) and were chosen by convenience sampling; thus, the findings might not be generalizable to other areas of China.

## Conclusions

The findings of this study suggest that children with behavioral disorders are more inclined to suffer from unintentional injuries. Behavioral disorders such as schizoid, anxiety/depression, hyperactivity and delinquent behavior could predict unintentional injury. The results support the importance of assessing behavioral characteristics among school-age children and highlight the necessity of doing interventions to assist both parents and their children in managing or reducing behavioral disorders and preventing unintentional injury. Pediatric care providers should learn behavior management strategies to reduce injury risk and seek effective methods to recognize children with behavior disorders for injury prevention efforts. Safety education classes should be offered in schools, and these classes should be adaptable to address the different characteristics of children’s behavior.

### Ethics approval and consent to participate

Ethics approval was obtained from Harbin Medical University (Ethics Committee of Harbin Medical University No.15HMUSCI062). The informed consents were obtained from all participants.

## Abbreviations

CBCL: The Achenbach Child Behavior Checklist
ADHD: Attention deficit hyperactivity disorder
OR: Odds Ratio
CI: Confidence Interval
SD: Standard deviation
